# Peritoneal Tuberculosis in an Immunocompetent, Unknown Risk Patient

**DOI:** 10.1155/2013/680763

**Published:** 2013-06-26

**Authors:** Yutaka Tomizawa, Emmanuelle B. Yecies, Fiona E. Craig, Adam Sohnen

**Affiliations:** ^1^Department of Medicine, University of Pittsburgh Medical Center Presbyterian Shadyside, 5230 Centre Avenue, Pittsburgh, PA 15213, USA; ^2^University of Pittsburgh Graduate School of Medicine, 3550 Terrace Street, Pittsburgh, PA 15213, USA; ^3^Department of Pathology, University of Pittsburgh Medical Center, 200 Lothrop Street, Pittsburgh, PA 15213, USA

## Abstract

A 36-year-old man with no significant past medical history presented with two-month abdominal distention, night sweats, and weight loss of 15 Ib. He had no known exposure to tuberculosis. PPD test was negative prior to the hospital admission. Physical examination was notable for new onset ascites, but no superficial lymphadenopathy or stigmata of chronic liver disease was found. CT scan demonstrated enlarged mesenteric lymph nodes, and prominent retroperitoneal lymph nodes along with moderate ascites and omental infiltration. Diagnostic paracentesis yielded WBC of 295/mm^3^, lymphocytic predominance (70%), and serum ascitic albumin gradient of 0.1, consistent with exudate. Both the ascitic culture and AFB smear were negative, and ascitic cytology revealed nonmalignant cells. Exploratory laparoscopy for excisional biopsy of mesenteric lymph nodes was performed. Pathologic findings revealed caseous granulomas with scattered multinucleated giant cells. Mesenteric lymph node tissue culture subsequently grew *Mycobacterium tuberculosis* complex and the diagnosis of peritoneal tuberculosis was confirmed. The patient was started on quadruple therapy. A couple of days after the antibiotics were started, the small bowel obstruction started to resolve with resumption of bowel movements and tolerance of oral intake. A week later, ascites stopped accumulating and fever was no longer noted. He has been well and continues to be under observation.

## 1. Introduction

Although most prevalent as a pulmonary disease, tuberculosis can affect any part of the body, including the peritoneum. Most often, extrapulmonary tuberculosis is the result of reactivation of latent disease established by hematogenous spread during primary pulmonary infection. In most cases, immunocompromising conditions predispose to peritoneal tuberculosis, and risk factors include liver cirrhosis, diabetes mellitus, use of systemic corticosteroids, HIV infection, and underlying malignancy [1]. The rationale of testing for latent tuberculosis infection is to identify individuals who are at increased risk for the development of tuberculosis. Purified protein derivative (PPD) test along with chest X-ray is a widely available screening method. Interpretation of negative PPD test raises a concern of the possibility of false negative, however no definite test exists to help determine between true negative and false negative. Treating patients with a negative PPD test could be considered based on the pretest probability, and this clinical decision should be based on symptoms, physical examination, and baseline chest X-ray [2, 3]. The likelihood of reactivation of latent disease is considered low in an asymptomatic patient with a negative PPD test along with normal baseline chest X-ray.

## 2. Case Presentation

**Figure 1:**
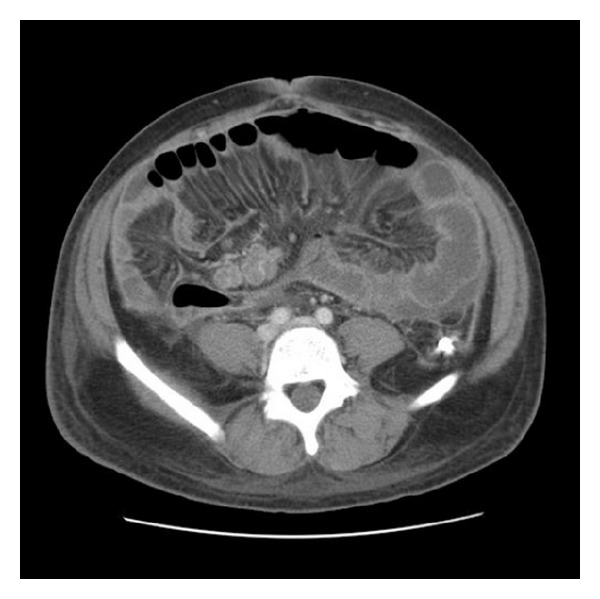
CT abdomen and pelvis revealing enlarged mesenteric lymph nodes and prominent retroperitoneal lymph nodes along with omental infiltration.

**Figure 2:**
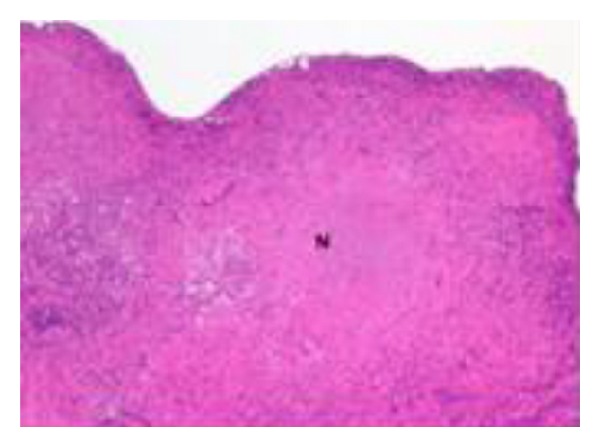
Mesenteric lymph node biopsy demonstrating granulomas with areas of caseous necrosis (N) (H&E stain, 10x magnification).

**Figure 3:**
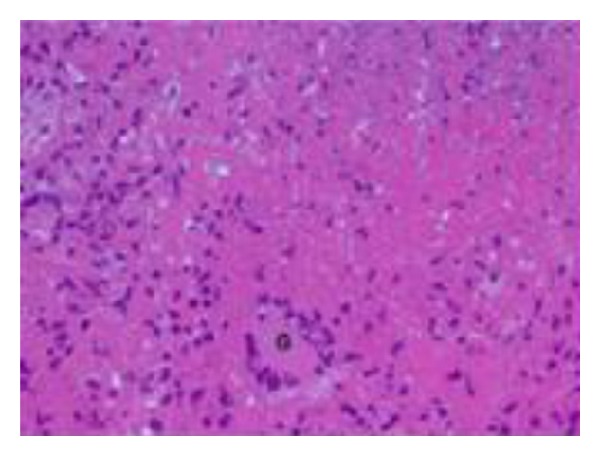
Scattered multinucleated giant cells (G) (H&E stain, 50x magnification).

**Figure 4:**
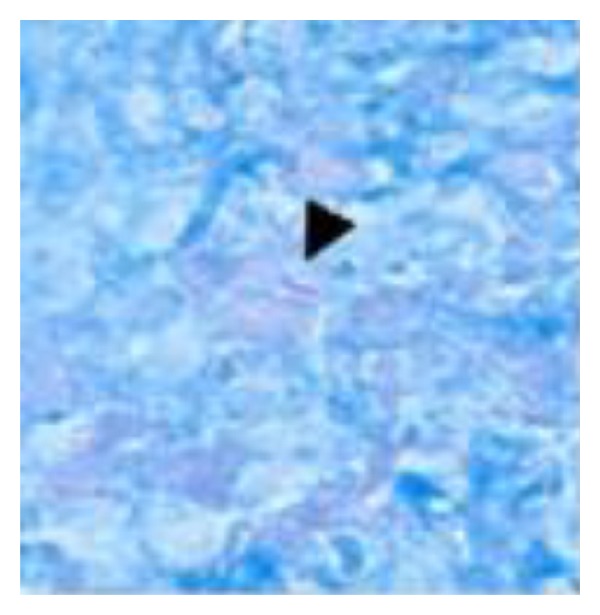
Acid fast bacilli (arrow head) (AFB stain, 100x magnification).

A 36-year-old man with no significant past medical history presented with two-month gradually worsening abdominal distention along with early satiety, night sweats, and weight loss of 15 Ib. He emigrated from Zambia 12 years ago and had not returned since arrival at the USA. He had never lived elsewhere. He had no known exposure to tuberculosis and no family history of tuberculosis. Chest X-ray was clear at the time of immigration and PPD test was negative three years prior to the hospital admission. Physical examination was notable for new onset ascites but no superficial lymphadenopathy or stigmata of chronic liver disease was found. Laboratory data were pertinent for WBC 2700 cells/mil (normal differential), serum total protein 7.7 g/dL, serum Alb 2.8 g/dL and serum LDH 245 IU/L. Liver function and coagulation tests were normal, and hepatitis B and C titers were negative. Chest X-ray was clear and did not show fibronodular changes. Abdominal X-ray showed distended small bowel loops. CT scan demonstrated enlarged mesenteric lymph nodes, prominent retroperitoneal, and pericardial lymph nodes along with moderate ascites with omental infiltration (Figure 1). Diagnostic paracentesis yielded clear yellow fluid with WBC of 295/mm^3^, lymphocytic predominance (70%), and serum ascetic albumin gradient of 0.1, consistent with exudate. It raised a concern for lymphoproliferative process such as malignant lymphoma. Both the ascitic culture and AFB smear were negative, and ascitic cytology revealed nonmalignant cells. Further serology tests were negative for monoclonal protein by serum protein electrophoresis, HIV-1/2 Ab, EBV-PCR, or CMV-PCR. Exploratory laparoscopy for excisional biopsy of mesenteric lymph nodes was performed. Pathologic findings revealed caseous granulomas (Figure 2) with scattered multinucleated giant cells (Figure 3). Acid-fast stains demonstrated several scattered rod-shaped acid-fast bacilli (Figure 4). Mesenteric lymph node tissue culture subsequently grew *Mycobacterium tuberculosis* complex. The diagnosis of peritoneal tuberculosis was confirmed. The patient was started on quadruple therapy (isoniazid, ethambutol, rifampin, and pyrazinamide). A couple of days after the antibiotics were started, the small bowel obstruction started to resolve with resumption of bowel movements and tolerance of oral intake. A week later, ascites stopped accumulating and fever was no longer noted. He has been well and continued under observation.

## 3. Discussion

We described a case of peritoneal tuberculosis in an immunocompetent patient who had no known risk factors and negative PPD test with clear chest X-ray. It was manifested by ascites, prominent mesenteric and retroperitoneal lymph nodes along with omental infiltration causing small bowel obstruction. Excisional biopsy of mesenteric lymph nodes confirmed the presence of caseous granuloma with scattered multinucleated giant cells and acid-fast stains demonstrated several scattered rod-shaped acid-fast bacilli. This case highlights that peritoneal tuberculosis should be considered in the differential diagnosis of lymphocytic ascites despite lack of suggestive risk factors or history.

Peritoneal tuberculosis can result from military tuberculosis, translocate transmurally from ingestion, or spread directly from adjacent organs. Prior studies suggest that only 33% of patients with peritoneal tuberculosis had evidence of primary pulmonary tuberculosis on chest X-ray. Though various risk factors were identified, 20%–33% of patients had no risk factors [1]. Progressive formation of tubercles throughout the peritoneum is the current understanding of the pathophysiology of peritoneal tuberculosis [4]. Clinical presentation is nonspecific and therefore hard to identify conclusively without diagnostic workup. Patients often present with ascites (90%), abdominal pain (80%–95%), fever (40%–70%), weight loss (40%–90%), and diarrhea (11%–20%). Small bowel obstruction has been linked to adhesion formation [5, 6]. Diagnosis is often difficult due to the lack of specificity in presentation and insidious onset. PPD test is only positive in 70% of cases and a negative result is of no help in excluding the disease [7]. Paracentesis most often demonstrates exudative, lymphocytic fluid. Diagnostic gold standard is the culture of Mycobacterium from ascitic fluid or peritoneal biopsy specimen. However, ascites culture and smear have low sensitivity (<20%) [7]. Laparoscopy can reveal the typical peritoneal studding of the tubercles and yield tissue for culture and smear. Visualization can be diagnostic in up to 95% of cases, and biopsies reveal caseating granulomas with almost 100% sensitivity [8, 9]. T-cell based testing for *Mycobacterium tuberculosis* (ELISPOT) has recently been approved by U.S. Food Drug Administration. This enzyme-linked immunospot assay measures gamma producing T-cell responses to early secreted antigenic targets of *Mycobacterium tuberculosis* and has shown promising results. A single study of 72 patients reported the sensitivity and specificity in the diagnosis of extrapulmonary tuberculosis as 94% and 88%, respectively, [10]. This assay on peripheral blood or ascitic fluid may prove to be a useful adjunct in the diagnosis of *Mycobacterium tuberculosis*; however it is not widely available. Treatment of confirmed peritoneal tuberculosis follows the principles of other forms of tuberculosis with quadruple therapy.

In conclusion, we reported a very unique case of peritoneal tuberculosis in an immunocompetent patient who had no known risk factors and a negative PPD test with clear chest X-ray. Further investigation is warranted to validate the diagnostic accuracy of noninvasive enzyme-linked immunospot assay test in the diagnosis of peritoneal tuberculosis. Until more sensitive diagnostic modalities are widely available, it is important for clinicians to maintain a high index of suspicion for peritoneal tuberculosis as a cause of new-onset lymphocytic ascites in an immunocompetent, unknown risk patient.

## References

[1] Kai MC, Chow VCY, Hung LCT, Shiu MW, Cheuk CS (2002). Tuberculous peritonitis-associated mortality is high among patients waiting for the results of mycobacterial cultures of ascitic fluid samples. *Clinical Infectious Diseases*.

[2] American Thoracic Society (2005). American Thoracic Society/Centers for Disease Control and Prevention/Infectious Diseases Society of America: controlling tuberculosis in the United States. *American Journal of Respiratory and Critical Care Medicine*.

[3] Gordin FM, Masur H (2012). Current approaches to tuberculosis in the United States. *The Journal of the American Medical Association*.

[4] Bhargava DK, Shriniwas S, Chopra P, Nijhawan S, Dasarathy S, Kushwaha AKS (1992). Peritoneal tuberculosis: laparoscopic patterns and its diagnostic accuracy. *American Journal of Gastroenterology*.

[5] Jakubowski A, Elwood RK, Enarson DA (1988). Clinical features of abdominal tuberculosis. *Journal of Infectious Diseases*.

[6] Demir K, Okten A, Kaymakoglu S (2001). Tuberculous peritonitis—reports of 26 cases, detailing diagnostic and therapeutic problems. *European Journal of Gastroenterology and Hepatology*.

[7] Marshall JB (1993). Tuberculosis of the gastrointestinal tract and peritoneum. *American Journal of Gastroenterology*.

[8] Manohar A, Simjee AE, Haffejee AA, Pettengell KE (1990). Symptoms and investigative findings in 145 patients with tuberculous peritonitis diagnosed by peritoneoscopy and biopsy over a five year period. *Gut*.

[9] Chow KM, Chow VC, Szeto CC (2003). Indication for peritoneal biopsy in tuberculous peritonitis. *American Journal of Surgery*.

[10] Kim S, Choi S, Kim H, Kim N, Oh M, Choe K (2007). Diagnostic usefulness of a T-cell-based assay for extrapulmonary tuberculosis. *Archives of Internal Medicine*.

